# Yeast cell factory: fishing for the best one or engineering it?

**DOI:** 10.1186/1475-2859-8-51

**Published:** 2009-10-12

**Authors:** Danilo Porro, Paola Branduardi

**Affiliations:** 1 University of Milano Bicocca, Department of Biotechnology and Biosciences, P.zza della Scienza 2, 20126 Milan, Italy

## Editorial

When today scientists and bioprocess engineers look at a **Microbial Cell Factory **for the production of a protein or a metabolite of commercial or research interest, they think at the microorganism of choice first from a (*i*) molecular, then from a (*ii*) metabolic and finally from a (*iii*) process point of view. Analyses and manipulations of the pathway(s) involved in the synthesis of the desired product and how this pathway(s) interacts with the overall cell functions and activities are indeed steps required to obtain high yield of the product (g of compound per g of substrate), high production (g/l) and high productivity (g/l/h). Conceptually, the whole set of biochemical reactions that take place in the microbial cell factory should be considered. This is valid for processes involving natural and/or recombinant products in wild type and/or engineered hosts.

Definitely, the whole approach was not straightforward when it was anticipated about 20 years ago by James Bailey, who proposed the development of a new discipline called Metabolic Engineering defined as "the improvement of cellular activities by manipulations of enzymatic, transport, and regulatory functions of the cell with the use of recombinant DNA technology" [1]. The development in genomic, molecular and bioinformatic tools together with high-throughput systems enormously speeded up the development of the existing as well as of new microbial cell factories.

Since 2002, Microbial Cell Factories has published more than 180 relevant manuscripts in form of Research Articles, Technical Notes, Reviews and Commentaries highlighting the role of hosting microbial cells for proteins and metabolites productions. About one fourth of all the manuscripts have the yeast *Saccharomyces cerevisiae *and other non-conventional yeasts as a subject. Many of these manuscripts are highly cited and, despite its young age, the Journal has become already a reference in the current yeast biotechnology literature.

A simple analysis of the manuscripts published during the last three decades clearly indicates a transition in the choice of the different yeast hosts. Indeed, starting from the early '80s, the majority of recombinant proteins produced in yeasts have been expressed using the conventional yeast *S. cerevisiae*. This was a direct reflection of the familiarity of molecular biologists with this yeast, combined with the deep knowledge about its genetics, biochemistry, physiology and fermentation technologies. Furthermore, *S. cerevisiae *is recognized by the American Food and Drug Administration (FDA) as an organism "generally regarded as safe" (GRAS). In this respect, it should be underlined that most of the recombinant pharmaceuticals so far approved for human use by the FDA and/or by the European Medicines Agency (EMEA) obtained by microbial eukaryotic cells have been obtained almost exclusively using *S. cerevisiae *[2]. However, it has to be said that sometimes this yeast is not the optimal host for large-scale production of heterologous proteins, especially because of its fermentation needs that require sophisticated equipment. In addition, the proteins produced by *S. cerevisiae *are often hyper-glycosylated and retention of the products within the periplasmic space, with a consequent partial degradation, is frequently observed. Disadvantages such as these have promoted, since the late '80s, a search for alternative hosts, trying to exploit the great biodiversity existing among the yeasts, and starting the development of expression systems using the so-called "non-conventional" yeasts.

Based on the manuscripts published by Microb Cell Fact, the most established alternative host used for the production of heterologous proteins is today the yeast *Pichia pastoris*, while *S. cerevisiae *is still the predominant host used for metabolite productions. Vaccines [3], receptors [4], industrial enzymes [5,6] and bacterial toxins [7], are among the most prominent compounds obtained by recombinant *P. pastoris *cell factories. The use of different promoters, culture medium and operational strategies for *P. pastoris *have been reviewed [8]. The analysis of transcriptional levels of the genes involved in protein synthesis and secretion is a key factor to understand the host organism's responses to recombinant protein production, as well as their interaction with the growth conditions. The transcriptional levels of some genes related to the unfolded protein response (UPR) and central metabolism have been analysed, revealing that overexpression and secretion of a recombinant lipase seems to trigger the UPR in *P. pastoris*, resulting in a physiological bottleneck for the production process [5]. However, when the genome sequence was not available, strain and process development relied mainly on analogies to other well studied yeasts like *S. cerevisiae *[9]. Finally, the genome of *P. pastoris *has been published [10,11]. A gene annotation  and an open access web based genome browser  are now available to the scientific community. A tremendous development of this host is also confirmed by many articles over viewing the development of new fermentation [3,4,12,13], scale-up [14] and modelling [15] strategies. The first example of a metabolite production in *P. pastoris *is related to the overexpression of the riboflavin biosynthetic pathway [16].

During the past years, great efforts have been dedicated to the development of yeasts fermenting xylose efficiently. Also in this respect, the *Pichia *genus has been an important source of hydrolytic enzymes, especially the ones involved in the xylose fermentation. The NAD(P)H-dependent *Pichia stipitis *xylose reductase (PsXR) is one of the key enzymes for xylose fermentation, and has been cloned into the commonly used ethanol-producing yeast *S. cerevisiae*. In order to eliminate the redox imbalance due to the preference of this enzyme for NADPH, successful strategies have been implemented to alter the coenzyme specificity [17]. Other non-conventional yeasts (*Hansenula polymorpha, Arxula adeninivorans *[18-22], *Candida boidinii *[22], *Pichia methanolica *[22], *Xanthophyllomyces dendrorhous *[23] and *Kluyveromyces lactis *[24,25] covered a less relevant space on Microb Cell Fact.

Going back to the prominent role covered by *S. cerevisiae*, a clear transition in the application fields has been observed. Indeed, the majority of the articles published in these last years by Microb Cell Fact are about the production of metabolites instead of heterologous proteins. The backer's yeast has long been utilized as a very efficient biocatalyst, thanks to its enzymatic capabilities, native or manipulated. Remarkably, it can be easily engineered to become able to produce small, huge and complex molecules, like a variety of flavonoids [26,27], isoprenoids and polyunsatured fatty acids [27], biofuels [28], fine chemicals and API (active pharmaceutical ingredient) [29], alkaloids [30] and organic acids [31]. In very recent years, many efforts have been devoted to widen the substrate range for *S. cerevisiae*. In particular, specific attention has been dedicate to make *S. cerevisiae *able to ferment xylose and/or arabinose [32-36] or to improve its growth on sucrose [37]. *S. cerevisiae *has been also the center of attention as a model for basic and applied virus research, including the analysis of the function of individual proteins from pathogenic viruses, the elucidation of key processes in viral replication and the use of this yeast in antiviral drug development and vaccine production [38]. In this respect, *S. cerevisiae *metabolome served as a tool to prioritize host targets [39]. Metabolite profiling has been also used for the characterization of the metabolic shift between oxidative and fermentative growth [40] as well as for the development of new technologies leading to yeast artificial chromosomes employed for random assembly of biosynthetic pathways [26]. A couple of articles have also been published about the modulation of the ribosomal subunit ratio [41] and regulation of gene expression [42] to maximize recombinant protein yield in *S. cerevisiae*, an enduring bottleneck in the post-genomic sciences.

Finally, an interesting comparative review of different microbial cell factories for recombinant protein production has considerably helped to describe the extremely rich landscape of in-vivo protein folding processes [43]. The effects of the composition of growth media in relation to strain development for industrial application and for heterologous protein and for bulk bio-commodity production have also been reviewed [44].

In the end, taking into consideration everything said so far, Microb Cell Fact articles show that manipulation of yeast cell factories for improving host properties and productions can be obtained by changing the external and/or the internal environment of the cell. Modulations of cell performances changing the external environment have long been and are still practiced by choosing selective operating conditions during the processes. Traditionally, internal changes have been and are still achieved by random mutagenesis, selection and, more directly, by means of rDNA approaches.

Based on my experience, I (DP) do believe that the best yeast cell factory could be obtained by fishing for the wild type or recombinant yeast cell factory having the best fitness for that specific production/process. I do also believe that it will be obtained by a deep analysis and following direct engineering of the whole set of biochemical reactions involved in the biosynthesis. Judging from the articles published by Microb Cell Fact, it seems clear that the first approach is more successful for the production of metabolites, while the second is for the production of proteins. At the same, we are both aware that selection remains the most powerful tool to catch the desired cell factory.

Concluding, the ideal solution would be a laboratory created biological system capable of replication and evolution, fed only by simple carbon and energy sources. The future is already under way: the chemical synthesis, assembly, and cloning of a bacterial genome in *S. cerevisiae *has been already described [45,46].
